# Probiotic Properties and Antioxidant Activities of *Pediococcus pentosaceus* SC28 and *Levilactobacillus brevis* KU15151 in Fermented Black Gamju

**DOI:** 10.3390/foods9091154

**Published:** 2020-08-21

**Authors:** Seo Jin Yang, Kee-Tae Kim, Tae Young Kim, Hyun-Dong Paik

**Affiliations:** 1 Department of Food Science and Biotechnology of Animal Resources, Konkuk University, Seoul 05029, Korea; vseojinv@hanmail.net (S.J.Y.); richard44@hanmail.net (K.-T.K.); 2 Food Research Center, Hansan Food & Grocery Co., Emseong-gun 27733, Korea; kty54090@naver.com

**Keywords:** antioxidant activity, gamju, immune-enhancing activity, *Aspergillus awamori*, lactic acid bacteria, probiotics

## Abstract

Black gamju is Korean traditional beverage fermented with molds. The aim of this study was to assess the probiotic properties and antioxidant activities of novel *Pediococcus pentosaceus* SC28 and *Levilactobacillus brevis* KU15151 to develop black gamju with bioactive properties for health. Tolerance against artificial gastric juice and bile salts, adhesion ability on HT-29 cells of strains, and antibiotics susceptibility were evaluated as probiotics, and various enzyme productions were detected. The 2,2-diphenyl-1-picrylhydrazyl assay, 2,2′-azinobis(3-ethylbenzothiazoline-6-sulfonate, and β-carotene bleaching assay were used for antioxidant activity of samples. The tolerance of both strains to artificial gastric juice and bile salts (Oxgall) was more than 90%. Additionally, both strains did not produce β-glucuronidase and were resistant to gentamicin, kanamycin, streptomycin, and ciprofloxacin. After fermentation of black gamju with each strain, the number of viable lactic acid bacteria increased to 8.25–8.95 log colony forming unit/mL, but the pH value of fermented samples decreased more (to pH 3.33–3.41) than that of control (pH 4.37). *L. brevis* KU15151 showed higher adhesion activity to HT-29 cells and antioxidant effects than *P. pentosaceus* SC28 in three antioxidant assays.

## 1. Introduction

Nowadays, consumers are increasingly demanding functional foods that are rich in probiotic bacteria [1,2]. Probiotics are defined as live microorganisms that, when administered in adequate amounts, confer a health benefit on the host such as immunomodulatory, antimicrobial, anticancer, and hypolipidemi effects [3]. In particular, they have been known to have antioxidant effects due to exopolysaccharides on cell wall; their antioxidant enzymes such as catalase, glutathione peroxidase, superoxide dismutase (SOD), catalase; various antioxidant compounds such as glutathione, tocopherol, and ascorbic acid [4]. Most lactic acid bacteria (LAB) and some *Bacillus* strains [5] have been used as probiotics and are generally recognized as safe [6]. *Lactobacillus*, *Leuconostoc*, *Streptococcus*, and *Pediococcus* are most popular as probiotic strains. However, as probiotics, LAB should safely pass through and attach to the gastrointestinal (GI) tract to have beneficial effects [7,8,9]. In addition, lactic acid fermentation should increase the nutritional value and prolong shelf life of food products [10]. The most commonly consumed probiotic foods are milk-based formulations such as yogurt and cheese. However, these milk-based formulations are not consumed by individuals with lactose intolerance and milk allergy and vegans [11]. In such cases, cereal-based probiotic beverages may be good alternatives to milk-based drinks [12].

Cereals are rich in carbohydrates and dietary fibers and provide minor nutrients such as minerals, vitamins, and phytochemicals [10]. In Asia, rice has long been used to make various beverages such as “bhaati jaanr” and “haria” (India) via fermentation [13]. During fermentation, mold, yeast [12], and lactic acid bacteria (LAB) [14,15] have been mainly used to enhance material digestibility and sensory properties and improve nutritional bio-functionality and shelf life [7]. Gamju (also called “sikhye”) is a traditional rice-based beverage in Korea, and it is usually served as a dessert. It is manufactured by mixing steamed rice, malt juice (wort), water, and a small amount of other ingredients such as ginger. During processing, α- and β-amylase in malt juice decompose the starch in steamed rice to maltose and glucose, and the sweetness of the product increases according to reaction time [16]. *Aspergillus awamori*, called ‘black mold’, is used to manufacture alcoholic beverages and traditional fermented seasoning [17], and it is generally cultured in cooked rice called “koji,” a type of seed culture. This strain can degrade raw starch in rice due to the production of variable exo-enzymes such as α-amylase, glucoamylase, and α-glucosidase [18], and it can improve the flavor of a malt juice during gamju processing due to its sweetness; this gamju is called “black gamju”.

Previous studies have focused on improving the physicochemical and sensorial properties of gamju by adding chestnut shell [17] or sweet pumpkin [19]. However, only a few studies have focused on evaluating the health benefits of gamju and improving gamju quality by making changes to processes such as fermentation. The lactic acid produced by LAB can contribute to the sour taste of black gamju to improve consumer acceptability.

Therefore, the aims of this study were to assess the probiotic characteristics of isolated LAB strains and to investigate the growth and antioxidant activities of LAB strains in black gamju product in vitro. These results may be helpful for developing new fermented cereal beverages in the food industry.

## 2. Materials and Methods

### 2.1. Isolation and Identification of Probiotic Strains

The LAB strains used in this study were isolated from traditional Korean food (octopus jeotgal and radish kimchi). One gram of each food sample was serially diluted and spread on de Man, Rogosa, and Sharpe (MRS; BD Biosciences, Franklin Lakes, NJ, USA) agar at 37 °C for 24 h. Then, every colony was inoculated and incubated in MRS broth at 37 °C for 24 h. Potential probiotic LAB strains were identified as *Pediococcus pentosaceus* SC28 and *Levilactobacillus brevis* KU15151 by using 16S rRNA sequencing performed by Bionics Inc. (Seoul, Korea). The results on sequencing were analyzed by comparing with GENBANK database using the Basic Local Alignment Search Tool (BLAST) website (http://blast.ncbi.nlm.nih.gov). As a reference probiotic strain, *Lactobacillus rhamnosus* GG was taken from the Korean Collection for Type Cultures (KCTC, Daejeon, Korea).

### 2.2. Tolerance to Artificial Gastric Juice and Bile Salts

The resistances of the isolated strains to artificial gastric juice and bile salts were evaluated as described by Guo et al. [20] with some modifications. The resistance to artificial gastric juice was measured after incubation in 50 mM sodium carbonate buffer including 0.3% (*w*/*v*) pepsin (Sigma-Aldrich, St. Louis, MO, USA), and the pH was controlled at 2.5 for 3 h at 37 °C. The resistance to bile salts was measured by incubation in MRS broth including 0.3% (*w*/*v*) Oxgall (BD Biosciences, Oxford, UK) at 37 °C for 24 h. The survival rate (%) of the strains was measured by enumerating the viable cells on the MRS agar plates.

### 2.3. Adhesion Ability to HT-29 Cells

In case of measurement of the adhesion ability of the LAB strains, HT-29 (KCLB 30038, a human colon adenocarcinoma cells) were used [21]. HT-29 cells (2 × 10^5^ cells/mL) were incubated in a 24-well plate at 37 °C for 24 h. The intact bacterial cells (1 × 10^7^ CFU/mL) of each LAB strain were added to the HT-29 cells and were incubated in Roswell Park Memorial Institute (RPMI) 1640 medium (Hyclone, Logan, UT, USA) at 37 °C for 2 h. Non-adherent bacterial cells were removed by washing three times with PBS. The adherent bacterial cells were detached using 1% Triton X-100 (Sigma-Aldrich, St. Louis, MO, USA) solution. Then, 100 μL of a detached cell broth was used for MRA plate counting. The adhesion ability was evaluated by counting the number of initial cells and adherent cells on the MRS plates as follows:

(1) $$\mathrm{The}\;\mathrm{adhesion}\;\mathrm{ability}\;\left(\%\right)=\left(\;\frac{\mathrm{Adherent}\;\mathrm{bacterial}\;\mathrm{cells}\;\left(\mathrm{CFU}/\mathrm{mL}\right)}{\mathrm{Initial}\;\mathrm{number}\;\mathrm{of}\;\mathrm{bacterial}\;\mathrm{cells}\;\left(\mathrm{CFU}/\mathrm{mL}\right)}\right)\times 100\%$$

### 2.4. Enzyme Production

To measure the various enzyme productions of LAB strains, the API ZYM kit (BioMerieux, Lyon, France) was used. The strains were suspended in phosphate buffered saline (PBS) (Gibco, Grand Island, NY, USA) at 10^6^ CFU/mL, added to each cupule, and incubated at 37 °C for 4 h. One drop of ZYM test reagent was inoculated, and the level of enzyme activity was determined as the degree of color change.

### 2.5. Antibiotic Susceptibility

The antibiotic susceptibility of the LAB strains was evaluated using the disk diffusion assay, according to the method of Yang et al. [21]. The culture solution (100 μL) of each LAB strain was spread onto MRS agar. Then, paper disks containing antibiotics were placed on the MRS agar and incubated at 37 °C for 24 h. The clear zone (mm) was measured and compared with the standards set by the Clinical and Laboratory Standards Institute (CLSI). Eight types of antibiotics were used in this test: ampicillin (0.2 g/L), chloramphenicol (0.6 g/L), ciprofloxacin (0.1 g/L), doxycycline (0.6 g/L), gentamicin (0.2 g/L), kanamycin (0.6 g/L), streptomycin (0.2 g/L), and tetracycline (0.6 g/L).

### 2.6. Black Gamju Fermentation and Investigation of Viable Cell Number and pH Value

Black gamju saccharified by *Aspergillus awamori* was obtained from Hansan F&G Co. (Eumseong, Korea). The black gamju was prepared as follows: black koji was made using *A. awamori*. Rice (10 kg) was steeped in distilled water at 20 °C for 2 h and steamed for 40 min. After cooling to 35 °C, the steamed rice was inoculated with 0.2% (*w*/*w*) *A. awamori* spores and incubated at 35 °C for 3 days. To produce black gamju, steeped rice, distilled water, and black koji were mixed at a ratio of 1:4:1 (weight) and incubated at 60 °C for saccharification. The saccharified black gamju was sterilized at 121 °C for 15 min.

*P. pentosaceus* SC28 and *L. brevis* KU15151 were used to ferment the black gamju. The fermented black gamju was prepared as follows: each LAB strain was separately inoculated into the black gamju at a final concentration of 10^5^ CFU/mL and fermented at 37 °C for 24 h in the incubator. Non-fermented black gamju was used as the negative control. The number of LAB strains in the black gamju was enumerated by counting viable cells on the MRS plates. The pH value of the fermented black gamju was measured using a pH meter (Model: pH7110, Xylem Analytics Germany GmbH, Weilheim, Germany).

### 2.7. Sample Preparation of Bacterial Cells and Extraction of Black Gamju Samples

LAB strains were cultured in MRS broth at 37 °C for 18 h. Intact cells were harvested by centrifuging (14,000× *g*) at 4 °C for 10 min. The harvested cells were washed three times and re-suspended in PBS (Summer Scientific, Waltham, MA, USA).

The fermented and non-fermented black gamju extracts were prepared as described by Ghosh et al. [22] as follows: the black gamju (50 mL) samples were extracted with 150 mL of methanol:acetone:water (4:3:3) mixture. The mixture was filtered with Whatman No. 2 filter paper and evaporated with a rotary vacuum evaporator (EYELAN-1000V; Tokyo, Japan) at 50 °C. The resultant products were lyophilized in a freezing dryer (Benchtop FDB; Operon, Gimpo, Korea) and stored at −18 °C until further used.

### 2.8. Antioxidant Activity of LAB Strains and Black Gamju Extracts

#### 2.8.1. 2,2-Diphenyl-1-picrylhydrazyl (DPPH) Radical Scavenging Activity

2,2-Diphenyl-1-picrylhydrazyl (DPPH) radical scavenging activity was evaluated as described by Das and Goyal [23] with some modifications. To evaluate the antioxidant activity of the LAB strains, 2 mL of DPPH solution (0.4 mM) in methanol and 2 mL of bacterial samples were mixed and incubated at 37 °C for 30 min in the dark. Ascorbic acid (1 mg/mL) was used as the positive control. The DPPH radical scavenging activity was calculated by measuring the absorbencies of the supernatant at 517 nm and using the following equation:

(2) $$\mathrm{DPPH}\;\mathrm{radical}\;\mathrm{scavenging}\;\mathrm{activity}\;\left(\%\right)=\left(1\;-\;\frac{A_{\;\mathrm{sample}}}{A_{\;\mathrm{control}}}\right)\times 100\%$$

where A_sample_ and A_control_ are the absorbance values of the bacterial sample and distilled water at 30 min, respectively.

#### 2.8.2. 2,2′-Azinobis(3-ethylbenzothiazoline-6-sulfonate) (ABTS) Radical Scavenging Activity 

2,2′-Azinobis(3-ethylbenzothiazoline-6-sulfonate) (ABTS) radical scavenging activity was evaluated as described by Verón et al. [24] with some modifications. To make the ABTS solution, 14 mM ABTS and 5 mM potassium persulfate dissolved in 0.1 M potassium phosphate buffer (pH 7.4) were mixed and diluted until that the absorbance at 734 nm was adjusted to 0.7 ± 0.02. One hundred and fifty microliters each of the bacterial samples, gamju extracts dissolved in distilled water, and 1 mg/mL ascorbic acid (positive control) were mixed with the same amount of ABTS solution and incubated at 37 °C for 10 min. Absorbance of the supernatant was measured at 734 nm and calculated using the following equation:

(3) $$\mathrm{ABTS}\;\mathrm{radical}\;\mathrm{scavenging}\;\mathrm{activity}\;\left(\%\right)=\left(1\;-\;\frac{A_{\;\mathrm{sample}}}{A_{\;\mathrm{control}}}\right)\times 100\%$$

where A_sample_ and A_control_ are the absorbance values of the sample (bacterial or gamju) and distilled water after reaction for 10 min, respectively.

#### 2.8.3. β-Carotene Bleaching Method

The β-carotene bleaching method was used as described by Kassim et al. [25] with some modifications. The β-carotene solution was composed of linoleic acid (132 μL), β-carotene (6 mg), and Tween 80 (600 μL) (Samchun Co., Seoul, Korea) with 20 mL of chloroform. The chloroform in the solution was removed using a rotary evaporator. The absorbance of solution at 470 nm was adjusted to 1.20 with distilled water. Two hundred microliters of bacterial samples, gamju extracts dissolved in distilled water (50 mg/mL), and 1 mg/mL ascorbic acid (positive control) was mixed with 4 mL of the solution and incubated at 50 °C for 2 h. The absorbance of the supernatant was measured at 470 nm for 0 and 2 h and calculated using the following equation:

(4) $$\mathrm{Inhibition}\;\mathrm{of}\;\beta -\mathrm{carotene}\;\mathrm{and}\;\mathrm{linoleic}\;\mathrm{acid}\;\mathrm{oxidation}\;\left(\%\right)=\frac{A_{\;\mathrm{sample},\;2\;h}\;-{\;A}_{\;\mathrm{control},\;2\;h}}{A_{\;\mathrm{control},\;0\;h}\;-{\;A}_{\;\mathrm{control},\;2\;h}}\times 100\%$$

where A_sample, 2 h_ and A_control, 2 h_ are absorbance values of the sample (bacterial or gamju) and distilled water after 2 h of the reaction, respectively. A_control, 0 h_ is the absorbance of distilled water at the initial time.

### 2.9. Statistical Analysis

All experiments were triplicated, the significant differences were determined using one-way analysis of variance (ANOVA), and Duncan’s multiple range tests were performed by using SPSS software (Version 24; SPSS, Inc., Chicago, IL, USA).

## 3. Results

### 3.1. Tolerance to Artificial Gastric Juice and Bile Salts

The tolerances to artificial gastric juice and bile salts of two strains were tested (Table 1). All the tested LAB strains showed more than 90% survival rate and no significant differences (*p* > 0.05) under acidic conditions. In contrast, significant differences (*p* < 0.001) were observed under basic conditions. The data showed that the survival rates of *L. rhamnosus* GG and *P. pentosaceus* SC28 against bile salts were 101.83% and 100.05%, respectively, whereas *L. brevis* KU15151 showed a tolerance of 97.96%. 

### 3.2. Adhesion Ability of LAB Strains to HT-29 Cells

To have beneficial effects on the host, LAB should be adherent and colonize the intestinal tissues of the host [26]. The adhesion ability of the strains to HT-29 cells is presented in Table 1. The adhesion ability of the tested strains appeared to be variable (<4.45–6.87%), depending on the LAB. *P. pentosaceus* SC28 and *L. rhamnosus* GG showed adhesion rates of 4.45% and 6.30%, respectively. *L. brevis* KU15151 had a higher adhesion rate of 6.87% than the other strains.

### 3.3. Enzyme Production by LAB Strains

In this study, production of bacterial enzymes was estimated as a biological property of LAB. The production of 19 enzymes by the LAB strains was evaluated using the API ZYM kit (Table 2). *P. pentosaceus* SC28 and *L. brevis* KU15151 did not produce β-glucuronidase, which is detrimental to the human intestine because it hydrolyzes glucuronides [27]. In addition, these LAB strains commonly produce acid phosphatase, β-galactosidase, leucine arylamidase, lipase, naphthol-AS-BI-phosphohydrolase, and valine arylamidase. In addition, *L. brevis* KU15151 produces cystine arylamidase, esterase, esterase lipase, α-galactosidase, α-glucosidase, and β-glucosidase. Lactose is hydrolyzed into glucose and galactose by β-galactosidase, which alleviates lactose intolerance [27].

### 3.4. Antibiotic Susceptibility of LAB Strains

As shown in Table 3, all the strains were resistant to four antibiotics, namely, gentamicin, kanamycin, streptomycin, and ciprofloxacin, but both *P. pentosaceus* SC28 and *L. brevis* KU15151 were sensitive to ampicillin, tetracycline, chloramphenicol, and doxycycline. 

### 3.5. Antioxidant Activity of LAB Strains

**Figure 1:**
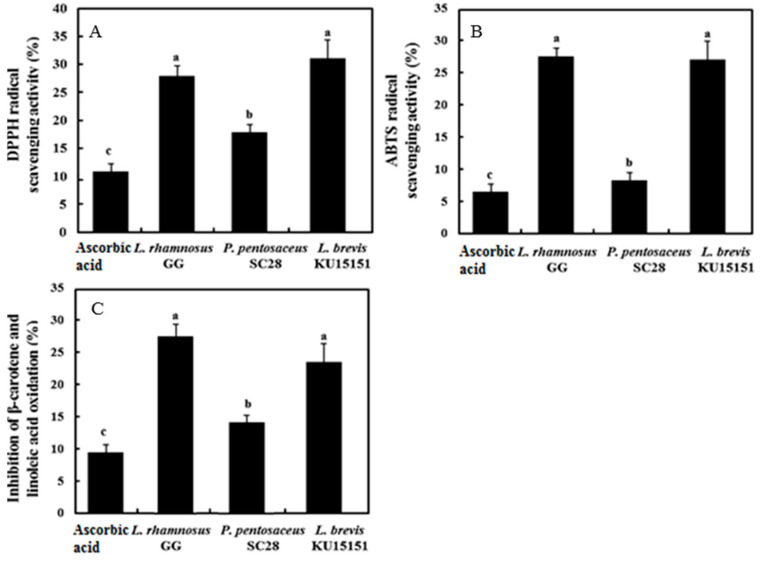
Antioxidant activities of LAB strains. (**A**) 2.2,-Diphenyl-1-picrylhydrazyl (DPPH) radical scavenging activity (%), (**B**) 2.2′-azinobis(3-ethylbenzothiazoline-6-sulfonate) (ABTS) radical scavenging activity (%), and (**C**) inhibition of β-carotene and linoleic acid oxidation (%). Different letters on each bar indicate significant differences between strain samples (*p* < 0.001).

In this study, three methods were performed to evaluate the antioxidant effects of the LAB strains (Figure 1). The DPPH radical scavenging assay results are presented in Figure 1A. The DPPH radical scavenging activity (%) was in the following order: *L. brevis* KU15151 (31.14%), *L. rhamnosus* GG (27.89%), and *P. pentosaceus* SC28 (17.83%). According to the ABTS radical scavenging assay (Figure 1B), the antioxidant activity of *L. brevis* KU15151 (27.50%) was similar to that of *L. rhamnosus* GG (27.55%) but greater than that of *P. pentosaceus* SC28 (8.65%). The β-carotene bleaching assay results are presented in Figure 1C. The inhibitory effects of *L. rhamnosus* GG and *L. brevis* KU15151 were 27.60% and 23.82%, respectively, whereas *P. pentosaceus* SC28 (14.18%) showed a lower inhibition rate. Thus, *P. pentosaceus* SC28 showed the lowest antioxidant effects, and *L. brevis* KU15151 was superior as an antioxidant.

### 3.6. Fermentation of Black Gamju Using LAB Strains

The samples of black gamju were separately fermented using *P. pentosaceus* SC28 and *L. brevis* KU15151. The viable cell numbers and pH values are presented in Table 4. The viable cell numbers in all fermented black gamju increased from 5 to 8.25–8.95 log CFU/mL for 1 day. Similarly, the pH value of fermented black gamju (pH 3.32–3.41) decreased when compared with non-fermented black gamju (pH 4.37). Due to the reaction of hydrolytic enzymes, the starch in rice was converted to sugars, and gamju contains high concentrations of glucose, maltose, and malto-oligomers [28]. The LAB strains were assumed to grow by consuming sugars in the gamju, and they produced organic acids such as lactic acid. 

### 3.7. Antioxidant Activity of Black Gamju Extracts

**Figure 2:**
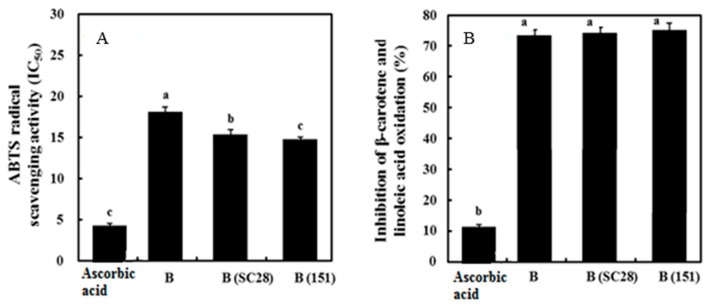
Antioxidant activity of black gamju extracts. (**A**) ABTS radical scavenging activity (IC_50_, mg/mL) and (**B**) inhibitory activity in β-carotene and linoleic acid oxidation (%). B, non-fermented black gamju extract; B (SC28), black gamju extract fermented by *Pediococcus pentosaceus* SC28; and B (151), black gamju extract fermented by *Levilactobacillus brevis* KU15151. Different letters on each bar present significant differences between values (*p* < 0.001).

The ABTS radical scavenging assay and β-carotene bleaching assay were conducted for evaluating the antioxidant potential of the black gamju extracts. As shown in Figure 2, IC_50_ values of non-fermented black gamju (B), black gamju fermented using *P. pentosaceus* SC28 (B (SC28)), and black gamju fermented using *L. brevis* KU15151 (B (151)) were 18.10, 15.33, and 14.74 mg/mL, respectively, and the radical scavenging effect against ABTS^+^∙ was increased in the black gamju extract fermented by *P. pentosaceus* SC28 and *L. brevis* KU15151.

The non-fermented and fermented black gamju extracts showed a high inhibition rate for β-carotene and linoleic acid oxidant. Especially, the inhibition rate of black gamju fermented using *L. brevis* KU15151 (75.49%) was higher than that of non-fermented black gamju (74.17%) and black gamju fermented using *P. pentosaceus* SC28 (74.57%). However, no significant differences (*p* > 0.05) were observed between the non-fermented black gamju and fermented black gamju extracts. Prevention of lipid peroxidation was not increased significantly by fermentation using LAB.

## 4. Discussion

The human stomach is an acidic environment in which the pH is 1.0–4.5 and ingested substances stay in the stomach for 3 h [29]. The intestinal tract is a basic environment in which 0.3% (*w*/*v*) bile salts are found [30]. Therefore, when the ingested LAB strains pass through the GI tract, the tolerance of the LAB strains to artificial gastric juice and bile salts is a very important factor. Some researchers have shown that the viable cell number of *P. pentosaceus* OZF decreased from 8.74 to 7.21 log CFU/mL when the strain was exposed to 0.3% oxgall for 4 h [31]. Bujnakova et al. [27] also reported that the survival rates of *Lactobacillus reuteri* L4/1 under acidic (0.3% pepsin, pH 2.5) and basic (0.3% oxgall, pH 7) conditions were 84% and 90%, respectively. Vidhyasagar et al. [26] reported that the cell number of potential probiotic *P. pentosaceus* VJ13 decreased to about 50% against gastric juice (pH 2 for 4 h). On the basis of our results, *P. pentosaceus* SC28 and *L. brevis* KU15151 could pass safely through the human GI tract because these two strains have superior tolerance to gastric acid and bile salts.

In addition, previous studies have reported that the adhesion ability to intestinal epithelium is related to hydrophobicity and auto-aggregation ability because the adhesion process involves interaction between the intestinal cells and bacterial cell surface [32,33,34]. Especially, Han et al. [32] and Bengoa et al. [33] reported that hydrophobic interaction allows bacterial cells and intestinal cells to bind more strongly and the auto-aggregation is related to charge and compounds such as proteins (SlpA) and exopolysaccharides of the LAB cell surface; *L. brevis* R4 and *L. acidophilus* AD1, which have the greatest hydrophobicity and auto-aggregation ability, have higher adhesion abilities to Caco-2 cells [32]. Jeon et al. [35] reported that 2–10% *Lactobacillus* strains can attach to the intestine. Potential probiotic *Lactobacillus plantarum* C182 showed 1.2% adhesion ability to HT-29 cells [36].

In this study, the adhesion ability of *P. pentosaceus* SC28 and *L. brevis* KU15151 to HT-29 cells showed that they have high adhesion and colonization ability for human intestine cells.

In this study, production of bacterial enzymes was estimated as a biological property of LAB. α-Glucosidase hydrolyzes disaccharides to glucose [37]. β-Glucosidase is used for the bioconversion of isoflavone, and substances produced by β-glucosidase have high absorption and bio-activity in the intestines [36]. In addition, *P. pentosaceus* SC28 and *L. brevis* KU15151 could be safe from the absence of β-glucuronidase. Generally, some probiotics that produce glucuronidase have been restricted because many toxic compounds (such as carcinogens) bound to glucuronic acid in the liver by phase II detoxification may be re-dissociated in the colon by glucuronidase and re-absorbed into the human body [38].

In this study, the susceptibility of LAB strains to various antibiotics was tested as a probiotic property. As probiotics, the strains that have beneficial effects on the health particularly of medicated patients should be resistant against various antibiotics. However, susceptibility to antibiotics may be different according to the LAB subspecies. According to Wang et al. [39], 10 *L. brevis* strains showed resistance to gentamicin, kanamycin, streptomycin, ciprofloxacin, and chloramphenicol. Some researchers reported that *P. pentosaceus* VJ13 was resistant to ampicillin, gentamicin, streptomycin, and ciprofloxacin and *P. pentosaceus* OZF was resistant to kanamycin [26,31]. In addition, the presence of antibiotic resistance determinants in probiotics has been concerned for safety because it is possible to be transferred to other pathogens in the human intestine by a genetic mechanism such as chromosomal recombination. In the intestine, bacteria containing antibiotic resistance genes may create other antibiotic-resistant bacteria [31]. According to the clinical and laboratory standards institute (CLSI) guideline, these strains were found to be safe for human health.

In previous studies, free radical scavenging activity, chelating activity, inhibitory activity of lipid peroxidation, and antioxidant-related enzyme activity were used to assess the antioxidant effects of LAB strains [32,40]. Han et al. [32] and Tang et al. [40] presented that the antioxidant substances of LAB strains are NADH, NADPH, antioxidant enzymes, Mn^2+^, bioactive compounds, and exopolysaccharides. Das and Goyal [23] found higher DPPH radical scavenging activity in *L. plantarum* DM5, *L. plantarum* B-4496, and *L. acidophilus* B-4495. Han et al. [32] reported that intact cells of LAB strains containing *P. pentosaceus* R1 and *L. brevis* R4 had significantly higher ABTS radical scavenging activity than cell-free extract and supernatant. Li et al. [41] showed that the free radical scavenging ability of intact cells is related to the cell surface materials of bacteria, e.g., proteins, polysaccharides, and lipoteichoic acid. In addition, Tang et al. [40] reported that the intact cells of *L. plantarum* MA2 prevented lipid peroxidation. On the basis of the results of the radical scavenging and β-carotene bleaching assays, *L. brevis* KU15151 showed superior antioxidant ability.

Various studies have reported non-dairy probiotic beverages. Chavan et al. [10] reported that non-dairy probiotic drinks that consist of cereal powder (barley, millet, and bean) and soymilk or almond milk showed an increase in acidity, decrease in pH, and increase in viable cell number. In addition, it was reported that a rice-based beverage fermented by *Lactobacillus fermentum* KKL1 showed a decrease in pH from 6.72 to 4.03 and increase in total titratable acidity from 0.01 to 0.84 and bacterial content [22].

Ghosh et al. [6] reported that haria, an Indian rice-based fermented beverage, showed higher DPPH radical scavenging activity as the fermentation time increased. In addition, the rice-based beverage fermented by *L. fermentum* KKL1 had a significantly higher amount of phenolic and flavonoid compounds and free radical scavenging ability [22]. These antioxidant effects were related to the higher concentration of oligosaccharides, flavonoids, and phenolic compounds in the fermented rice. On the basis of the antioxidant activities, the fermented black gamju has superior antioxidant ability than non-fermented black gamju. Therefore, the fermented black gamju could be used as a source of probiotic drinks.

## 5. Conclusions

*P. pentosaceus* SC28 and *L. brevis* KU15151 isolated from Korean traditional foods showed higher gastric acid and bile salts tolerances and adhesion abilities on HT-29 cells. Additionally, both *P. pentosaceus* SC28 and *L. brevis* KU15151 are safe because they do not produce β-glucuronidase, which causes toxin re-cycling in the human body, and have good antibiotic resistance. *L. brevis* KU15151 exhibited greater radical scavenging activity and lipid peroxidation inhibitory activity than *P. pentosaceus* SC28. After the black gamju was inoculated with the LAB strains, the viable cell number increased, and pH value decreased when compared with non-fermented black gamju. Furthermore, the fermented black gamju extracts showed higher antioxidant activities than the non-fermented black gamju extract except for β-carotene and linoleic acid anti-oxidation. Therefore, *P. pentosaceus* SC28 and *L. brevis* KU15151 can be used as potential probiotics, and the fermented black gamju can be used as a probiotic drink.

## Figures and Tables

**Table 1:** Artificial gastric juice and bile salt tolerance (%) and adhesion ability (%) of lactic acid bacteria (LAB) strains.

LAB Strains	Survival Rate (%)		Adhesion Ability (%)
	Gastric Acid Tolerance (0.3% Pepsin, pH 2.5)	Bile Salt Tolerance (0.3% Oxgall)	
*L. rhamnosus* GG	96.69 ± 1.04	101.83 ± 0.87 ^a^	6.30 ± 0.51 ^a^
*P. pentosaceus* SC28	96.37 ± 3.02	100.05 ± 0.22 ^b^	4.45 ± 0.19 ^b^
*L. brevis* KU15151	94.52 ± 2.87	97.96 ± 0.37 ^c^	6.87 ± 0.35 ^a^

^a, b, c^ Different superscript letters in the same column mean significant differences in each characteristic (*p* < 0.001). All values present as mean ± standard deviation of triplicate experiments.

**Table 2:** Analysis of enzyme production by two strains with the API ZYM kit.

Enzymes	Enzyme Activity ^(1)^	
	*P. pentosaceus* SC28	*L. brevis* KU15151
Control	0	0
*N*-Acetyl-β-glucosaminidase	0	0
Acid phosphatase	1	1
Alkaline phosphate	0	0
α-Chymotrypsin	0	0
Cystine arylamidase	0	1
Esterase	0	1
Esterase lipase	0	1
α-Fucosidase	0	0
α-Galactosidase	0	1
β-Galactosidase	1	3
α-Glucosidase	0	1
β-Glucosidase	0	3
β-Glucuronidase	0	0
α-Mannosidase	0	0
Naphthol-AS-BI-phosphohydrolase	1	1
Valine arylamidase	3	2

^(1)^ 0, 0 nM; 1, 5 nM; 2, 10 nM; 3, 20 nM; 4, 30 nM; 5, ≥40 nM.

**Table 3:** Antibiotic susceptibility of LAB strains.

Antibiotics	LAB Strains	
	*P. pentosaceus* SC28	*L. brevis* KU15151
Ampicillin	S ^(1)^	S
Chloramphenicol	S	S
Ciprofloxacin	R	R
Doxycycline	S	S
Gentamicin	R	R
Kanamycin	R	R
Streptomycin	R	R
Tetracycline	S	S

^(1)^ S, susceptible (over than 5 mm); I, intermediate (0–5 mm); R, resistant (not detected).

**Table 4:** Viable cell number and pH value of non-fermented and fermented black gamju.

Sample ^(1)^	Viable Cell Number (log CFU/mL)	pH
B	–	4.37 ± 0.02 ^a^
B (SC28)	8.25 ± 0.08 ^b (2)^	3.41 ± 0.02 ^b^
B (151)	8.95 ± 0.01 ^a^	3.33 ± 0.01 ^c^

^(1)^ B, non-fermented black gamju; B (SC28), black gamju fermented by *P. pentosaceus* SC28; B (151), black gamju fermented by *L. brevis* KU15151; ^(2) a–c^ Different superscript letters in the same column present significant differences in each characteristic (*p* < 0.001). All values are mean ± standard deviation (SD) of triplicated experiments.
